# Using sheep genomes from diverse U.S. breeds to identify missense variants in genes affecting fecundity

**DOI:** 10.12688/f1000research.12216.1

**Published:** 2017-08-02

**Authors:** Michael P. Heaton, Timothy P.L. Smith, Bradley A. Freking, Aspen M. Workman, Gary L. Bennett, Jacky K. Carnahan, Theodore S. Kalbfleisch

**Affiliations:** 1 U.S. Meat Animal Research Center (USMARC), Clay Center, NE, 68933, USA; 2 Department of Biochemistry and Molecular Biology, School of Medicine, University of Louisville, Louisville, KY, 40202, USA

**Keywords:** Sheep, Whole genome sequence, GDF9, BMP15, BMP1RB, Fecundity, Litter size

## Abstract

*Background*:  Access to sheep genome sequences significantly improves the chances of identifying genes that may influence the health, welfare, and productivity of these animals.

*Methods*:  A public, searchable DNA sequence resource for U.S. sheep was created with whole genome sequence (WGS) of 96 rams.  The animals shared minimal pedigree relationships and represent nine popular U.S. breeds and a composite line.  The genomes are viewable online with the user-friendly Integrated Genome Viewer environment, and may be used to identify and decode gene variants present in U.S. sheep.

*Results*:  The genomes had a combined average read depth of 16, and an average WGS genotype scoring rate and accuracy exceeding 99%.  The utility of this resource was illustrated by characterizing three genes with 14 known coding variants affecting litter size in global sheep populations:  growth and differentiation factor 9 ( *GDF9), *bone morphogenetic protein 15 ( *BMP15*), and bone morphogenetic protein receptor 1B ( *BMPR1B*).  In the 96 U.S. rams, nine missense variants encoding 11 protein variants were identified.  However, only one was previously reported to affect litter size ( *GDF9* V371M, Finnsheep).  Two missense variants in *BMP15* were identified that had not previously been reported:  R67Q in Dorset, and L252P in Dorper and White Dorper breeds. Also, two novel missense variants were identified in *BMPR1B*:  M64I in Katahdin, and T345N in Romanov and Finnsheep breeds.  Based on the strict conservation of amino acid residues across placental mammals, the four variants encoded by *BMP15 *and *BMPR1B* are predicted to interfere with their function.  However, preliminary analyses of litter sizes in small samples did not reveal a correlation with variants in *BMP15* and *BMPR1B* with daughters of these rams.

*Conclusions*: Collectively, this report describes a new resource for discovering protein variants *in silico* and identifies alleles for further testing of their effects on litter size in U.S. breeds.

## Introduction

There are currently 48 Mendelian traits and disorders in sheep where the causative variants are known ^1^. Many of these variants affect the gene’s protein sequence, and thereby alter its normal function. Although gene function may be affected by a wide range of large and small scale genomic sequence differences ^2, 3^, variants that alter amino acid sequences via missense, nonsense, frameshift, and splice site variants, are among those most likely to affect function ^4^. DNA polymorphisms encoding these protein variants are readily identified by aligning genomic sequences of animals to a high-quality, annotated reference genome assembly like that available for sheep ^3^. Identifying protein variants encoded by individuals in a population is an essential first step in characterizing genes known to influence traits ^5, 6^.

In principle, protein variants may be identified *in silico* for a gene of interest with access to population-scale whole genome sequence (WGS) data, like that found at the National Center for Biotechnology Information (NCBI) BioProjects and Sequence Read Archives (SRA). The first large ovine BioProject was deposited by the International Sheep Genomics Consortium (ISGC), which included the genome sequences of 75 sheep from 43 breed groups and two wild species from around the world ( PRJNA160933). Although global diversity is outstanding in these sheep, these animals are not ideally suited for protein variant discovery across U.S. sheep populations due to their exotic breed composition and low numbers within breed. In addition, the terabyte size of SRA datasets is challenging to work with, and not readily searchable by gene or accessible on the internet with a user-friendly environment, such as the Integrated Genome Viewer (IGV) ^7, 8^.

We previously showed in cattle that protein variants for a gene of interest may be identified *in silico* with the appropriate population sample and 14x WGS datasets ^9^. To that end, we created a similar publicly accessible, 16x WGS resource of 96 rams, that is viewable online with IGV. The rams share minimal pedigree relationships, and represent nine popular U.S. breeds and a composite line. Their genomes may be used to identify DNA polymorphisms in genes that affect the protein sequences in U.S. sheep populations. To highlight the utility of this resource, we analyzed three well-studied genes previously shown to encode protein variants affecting litter size in sheep: growth and differentiation factor 9 ( *GDF9),* bone morphogenetic protein 15 ( *BMP15*), ** and bone morphogenetic protein receptor 1B ( *BMPR1B*). Together, there are 14 previously reported missense, nonsense, and frameshift variants affecting the protein function of these genes, and thereby affect ovulation rate and litter size ^10, 11^.

The proteins encoded by *GDF9* and *BMP15* are oocyte-secreted paralogs of the transforming growth factor-beta (TGF-β) superfamily that form homo- and heterodimeric ligands, and are essential for ovarian and follicular development ^12^. These ligands synergistically regulate folliculogenesis through complex interactions with multiple receptors, such as BMPR1B. The *BMPR1B* gene encodes a type 1 membrane protein receptor that binds GDF9 and BMP15 in some mammals, although the identities of the BMPR1B ligands in sheep are unknown ^13^. The amino acid sequences of GDF9, BMP15, and BMPR1B are highly conserved among placental mammals, and variants that alter key residues in peptide sequence, diminish function, and affect traits like ovulation rate and litter size. For example, substitution of arginine (R) for glutamine (Q) at position 249 (Q249R) in *BMPR1B* causes attenuation of BMPR1B signaling and ultimately leads to an increase ovulation rate ^14, 15^. Likewise, missense, nonsense, and frameshift variants in *GDF9* and *BMP15* may abolish function and cause an increase in ovulation rate in carrier ewes, while causing sterility in homozygous ewes ^10^. However, some homozygous missense variants only diminish the protein’s biological activity. For example, the homozygous substitution of methionine (M) for valine (V) at position 371 (V371M) in *GDF9* allows ewes to remain fertile and hyper prolific. Since the types and distribution of protein variants encoded by these genes was unknown in U.S. sheep, we sought to identify them with WGS from the set of 96 U.S. rams.

We identified nine missense variants and 11 encoded protein variants in the three genes evaluated. Only one variant was previously known to be associated with increased litter size ( *GDF9*, V371M). However, four variants were not previously reported. In *BMP15*, a Q for R substitution was observed at position 67 (R67Q), and a proline (P) for leucine (L) substitution was observed at position 252 (L252P). In *BMPR1B*, an isoleucine (I) for M at position 64 (M64I), and an asparagine (N) for threonine (T) was observed at position 345 (T345N). Based on the pattern of evolutionary conservation for these residues in vertebrates, it was hypothesized that some of these novel missense variants could interfere with protein function, affect litter size, and be useful for producers interested in modulating lamb production to match available resources.

## Methods

### Ethics statement

This article contains no studies performed with animal subjects. The archival DNA samples used were collected between the years 2000 and 2006 ^16^. The reproduction records used were from daughters born between 2001 and 2007. All animal procedures were reviewed and approved by the United States Department of Agriculture (USDA), Agricultural Research Service (ARS), U.S. Meat Animal Research Center (USMARC) Animal Care and Use Committee prior to their implementation (Experiment Number 5438-31000-037-04). Because health status is important for providing purified DNAs to an international community as described here, tissues were collected from healthy sheep, without signs or history of clinical disease. The source flock’s history of disease surveillance is also relevant when requesting reference samples described in this report. Since first stocking sheep in 1966, USMARC has not had a known case of scrapie. Until 2002, surveillance consisted of monitoring sheep for possible signs of scrapie and submitting brain samples to the USDA Animal and Plant Health Inspection Service (APHIS) National Veterinary Services Laboratory in Ames, IA for testing. All tests have been negative. Since April 2002, USMARC has voluntarily participated in the APHIS Scrapie Flock Certification Program, is in compliance with the National Scrapie Eradication Program, and is certified as scrapie-free. With regards to other transmissible diseases, it is recognized that the USMARC flock of 2000 to 4000 breeding ewes is located in a bluetongue medium incidence area and is known to have some prevalence of contagious ecthyma (sore mouth), foot rot, paratuberculosis (Johne's disease), ovine progressive pneumonia (visna-maedi), and pseudotuberculosis caseous lymphadenitis.

### Design of the sheep diversity panel

**Figure 1.**
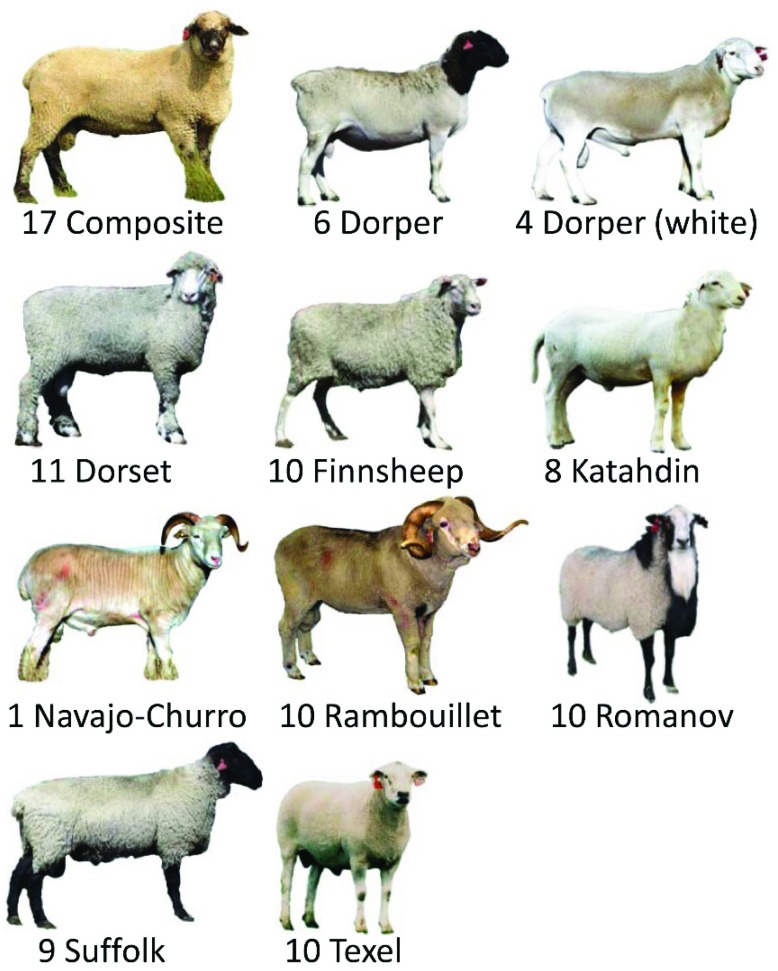
USMARC Sheep Diversity Panel version 2.4. This group of 96 rams was sampled from USMARC and private U.S. flocks to represent genetic diversity for traits such as fertility, prolificacy, maternal ability, growth rate, carcass leanness, wool quality, mature weight, and longevity.

The purpose of the USMARC Sheep Diversity Panel version 2.4 (MSDPv2.4) was to provide a set of 96 samples for variant allele discovery and frequency estimation in U.S. sheep. Details of the panel design strategy have been published elsewhere ^16^. Briefly, the panel consists of 96 rams from Dorper, White Dorper, Dorset, Finnsheep, Katahdin, Rambouillet, Romanov, Suffolk, and Texel breeds; a composite line (USMARC III: 1/2 Columbia, 1/4 Hampshire, and 1/4 Suffolk ^17^); and one Navajo-Churro ram ( Figure 1). In addition to their contributions to the U.S. sheep industry, the breeds were selected to represent genetic diversity for traits such as fertility, prolificacy, maternal ability, growth rate, carcass leanness, wool quality, mature weight, and longevity. The Navajo-Churro ram was included for its rare lysine 171 (K171) substitution in the prion gene. The rams sampled from each breed were chosen to minimize their genetic relationships at the grandparent level. DNA samples of all 96 rams have been made available for global use as genotyping reference material since 2010 ^16^.

### WGS production, alignment, and SNP genotyping

DNA was extracted from whole blood with a typical phenol:chloroform method and stored at 4°C in 10 mM TrisCl, 1 mM EDTA (pH 8.0) as previously described ^16^. Library preparation for DNA sequencing was also accomplished as previously described ^9^. Briefly, 2 μg of ovine genomic DNA was fragmented and used to make indexed, 500 bp, paired-end libraries. Pooled libraries were sequenced with a massively parallel sequencing machine and high-output kits (NextSeq500, two by 150 paired-end reads, Illumina Inc.). Pooled libraries with compatible indexes were repeatedly sequenced until 40 GB of data with greater than Q20 quality was collected for each ram, thereby producing at least 10-fold mapped read coverage for each index. This level of coverage provides scoring rates and accuracies that exceed 99% ^9, 18^. The DNA sequence alignment process was similar to that previously reported ^18^. FASTQ files were aggregated for each animal and DNA sequences, aligned individually to Oar_v3.1 with the Burrows-Wheeler Alignment tool (BWA) aln algorithm version 0.7.12 ^19^, merged, and collated with the bwa sampe command. The resulting sequence alignment map (SAM) files were converted to binary alignment map (BAM) files, and subsequently sorted via SAMtools version 1.3.1 ^20^. Potential PCR duplicates were marked in the BAM files using the Genome Analysis Toolkit (GATK) version 3.6 ^21^. Regions in the mapped dataset that would benefit from realignment due to small indels were identified with the GATK module RealignerTargetCreator, and realigned using the module IndelRealigner. The BAM files produced at each of these steps were indexed using SAMtools. The resulting indexed BAM files were made immediately available via the Intrepid Bioinformatics genome browser with groups of animals linked at the USDA, ARS, USMARC internet site.

The raw reads were deposited at NCBI BioProject PRJNA324837. Mapped datasets for each animal were individually genotyped with the GATK UnifiedGenotyper with arguments “--alleles” set to the VCF file ( Supplementary File S1), “--genotyping_mode” set to “GENOTYPE_GIVEN_ALLELES”, and “--output_mode” set to “EMIT_ALL_SITES”. Lastly, some SNP variants were identified manually by inspecting the target sequence with IGV software version 2.1.28 ^7, 8^ (described below in Methods section entitled ‘Identifying protein variants encoded by *GDF9, BMP15, and BMPR1B* genes’). In these cases, read depth, allele count, allele position in the read, and quality score were taken into account when the manual genotype determination was made.

### Evaluating WGS data integrity with 163 reference SNPs and 50 k bead array SNPs

Genotypes from a set of 163 reference SNPs were used as an initial verification of the WGS datasets. These DNA markers have been used for parentage determination, animal identification, and disease traceback ^22^. The 163 reference SNPs were previously genotyped across the MSDPv2.4 by multiple overlapping PCR-Sanger sequencing reactions, multiplexed matrix-assisted laser desorption/ionization time-of-flight mass spectrometry (MALDI-TOF MS) genotyping assays, and 50 k bead array platforms ^22^. The genotype call rate was defined as the number of SNP sites with three or more mapped reads, divided by the total number of sites tested. The error rate in the WGS data was estimated by comparing the independently-derived consensus genotypes for these SNPs to the WGS genotypes. An animal’s WGS dataset passed initial verification when the accuracy of the WGS genotypes exceeded 97%, and the average mapped read depth was proportional to the amount of WGS data collected. Animals’ datasets that failed this initial verification were inspected for contaminating and/or missing files. Once identified, the dataset was corrected and reprocessed. Linear regression analysis was accomplished in Excel version 2016. Access to the sequence was made available via USDA, ARS, USMARC internet site. Because the raw datasets were available online as they were produced, the raw FASTQ files were deposited in the NCBI SRA only after they were validated as described above. These 96 sets of files may be accessed through BioProject PRJNA324837 in the Project Data table under the Resource Name: SRA Experiments.

SNPs from the OvineSNP50 BeadChip (Illumina Inc.) were selected for comparison because they were numerous, uniformly distributed across the ovine genome, and available. Based on the nucleotide sequence of the 54,242 probes obtained from the manufacturer, the positions of 51,796 SNPs were verified via a BLAT process, as previously described ^18^. There were 50,357 of these that mapped uniquely to autosomes and were used for analysis ( Supplementary File S1). The genotypes from the WGS data were compared to those from the 50 k bead array with a custom program written specifically for this operation.

### Identifying protein variants encoded by GDF9, BMP15, and BMPR1B genes

The nucleotide variation in the exon regions of *GDF9, BMP15,* and *BMPR1B* was visualized through the public access portal at ARS USMARC with open source software installed on a laptop computer. Variants were recorded manually in a spreadsheet as previously described ^9^. Briefly, a Java Runtime Environment version 8, update 131 (Oracle Corporation, Redwood Shores, CA) was first installed on the computer. When links to the data were selected from the appropriate web page, IGV software version 2.1.28 ^7, 8^ automatically loaded from a third-party site (University of Louisville, Louisville KY) and the mapped reads were loaded in the context of the ovine Oar_v3.1 reference genome assembly. Gene variants were viewed by loading WGS from a set of eight animals of different breeds, and the IGV browser was directed to the appropriate genome region by entering the gene abbreviation in the search field (e.g., GDF9). The IGV zoom function was used to view the first exon at nucleotide resolution with the “Show translation” option selected in IGV. Since *GDF9* was in the reverse orientation with regards to the Oar_v3.1 assembly, the reference sequence was reversed so the translation was correctly viewed from right to left. The exon sequences were visually scanned for polymorphisms that would alter amino acid sequences, such as missense, nonsense, frameshift, and splice site variants. Once identified, the nucleotide position corresponding to a protein variant was viewed and recorded for all 96 animals. Using IGV, codon tables, and knowledge of the ovine GDF9, BMP15, and BMPR1B protein sequences ( NP_001136360.2, NP_001108239.1, and NP_001009431.1, respectively), the codons affected by nucleotide alleles were translated into their corresponding amino acids and their Oar_v3.1 positions noted. Haplotype-phased protein variants were unambiguously assigned in individuals that were either: 1) homozygous for all variant sites, or 2) had exactly one heterozygous variant site. Maximum parsimony phylogenetic trees were manually constructed from the unambiguously phased protein variants. The phylogenetic trees were used, together with simple maximum parsimony assumptions, to infer haplotype phase in seven rams where two heterozygous variant sites occurred in *GDF9*. The protein phylogenetic trees were rooted by comparing the variable residues in sheep to those from related species. Ovine peptide sequences for GDF9, BMP15, and BMPR1B were used to search NCBI's refseq_protein database with BLASTP 2.6.1 ^23, 24^. Aligned protein sequences from a representative subset of 29 vertebrate species were used for the comparison.

### Statistical analysis of litter size in daughters of carrier rams

Lambing records for daughters of carrier rams were retrieved from the USMARC historical database and analyzed with the mixed-model analysis of variance procedure (MIXED) of SAS (SAS Inst., Inc., Cary, NC; version 9.3). The phenotype evaluated was total number of lambs born (including stillborn) as a repeated record for each ewe. Different sets of ewes contributed to the analysis of each gene locus, and breed-specific genotype contrasts were evaluated. There were, however, similar models employed for all of the analyses. The models included fixed effects of classification for ewe age, and the sire-derived genotype class for the allele contrast in question. Three groups were created for ewe age to combine similar biologically performing ages: Group 1, ewe lambs; Group 2, ewes aged 2–5 years; and Group 3, ewes older than 5 years. The random effect of “ewe” was fitted and used to test the genotype contrast mean square. The Kenward-Roger option was used to approximate denominator degrees of freedom associated with the random effect of “ewe”. For analysis of the X-linked *BMP15* allele contrasts, the sire-derived gamete in these daughters was known directly. For analyses of autosomal genotype contrasts, it was inferred that rams of different genotypes had different distributions of daughter genotypes sampled. This inference reduced the power of analysis compared to a direct allelic test because we cannot determine the maternal-derived allele.

## Results

### Genome sequencing and validation of WGS datasets

**Figure 2.**
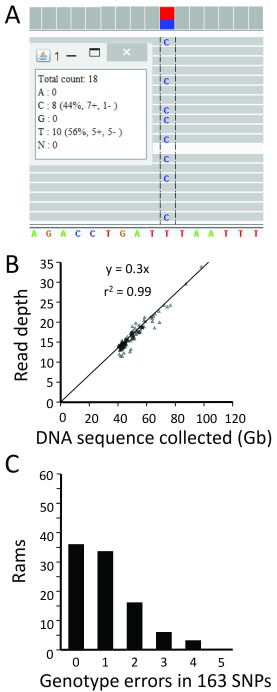
Comparison of 163 reference SNP genotypes with those derived from WGS data. ( **A**) Computer screen image of one animal’s WGS data aligned to ovine reference assembly Oar_v3.1 at a reference SNP site. The heterozygous C/T genotype is shown as viewed with the IGV software ^7, 8^. ( **B**) Linear relationship between mapped read depth and the amount (Gb) of Q20 WGS data collected. At each SNP position, the read depth and genotypes were visualized and manually recorded for 163 parentage SNPs. ( **C**), genotype scoring accuracy for 163 parentage SNPs in 96 sires. Consensus reference genotypes (n = 15,684) for the parentage SNPs were previously determined by multiple methods ^22^.

The average amount of genomic DNA sequence collected per animal was 50.4 GB (range 40.0 - 97.7, SD 10.4). Independently-derived genotypes from two sets of reference SNPs were used to confirm the identity and evaluate the quality of these data: those from 163 parentage markers, and those from approximately 50,000 markers on the OvineSNP50 bead array. Both sets have SNPs that are well distributed, highly-informative, and have been widely used. The WGS-derived genotypes for the 163 parentage SNPs were obtained by manually viewing an animal’s mapped reads at the relevant genome coordinates via the internet and third-party software (illustrated in Figure 2A, and described in Methods). The expected genotypes and read depths were consistent for all but one of the 96 datasets, owing to missing data for that animal. After rectifying the data omission and performing regression analysis of the data for all 96 rams, the average calculated read depth (17.0) was directly proportional to the amount of sequence collected for each animal (range 11.9 - 33.9, SD 3.6; Figure 2B).

The genotype call rate for the 163 parentage markers was 99.7% when WGS data was used, i.e. 47 missing of 15,159 possible. Most of the missing genotypes (32) were attributed to a single SNP site (DU191809, chr1:187087905). The source of the difficulty appeared to be a misassembly of the Oar_v3.1 in that that region, leading to a mismapping of reads as this site averaged only 3.5 reads per animal. The overall accuracy of WGS genotypes for the 163 reference SNPs was 99.4%, and no animals had a SNP genotype accuracy less than 97% (i.e., not more than 4 errors in 163 SNP genotypes; Figure 2C). The few WGS genotype errors observed were typically caused by undetected heterozygous alleles at sites with low read coverage. Thus, comparing genotypes from 163 reference SNPs to those derived from the WGS file sets was effective for discovering and repairing errors, and independently verifying coverage.

**Figure 3.**
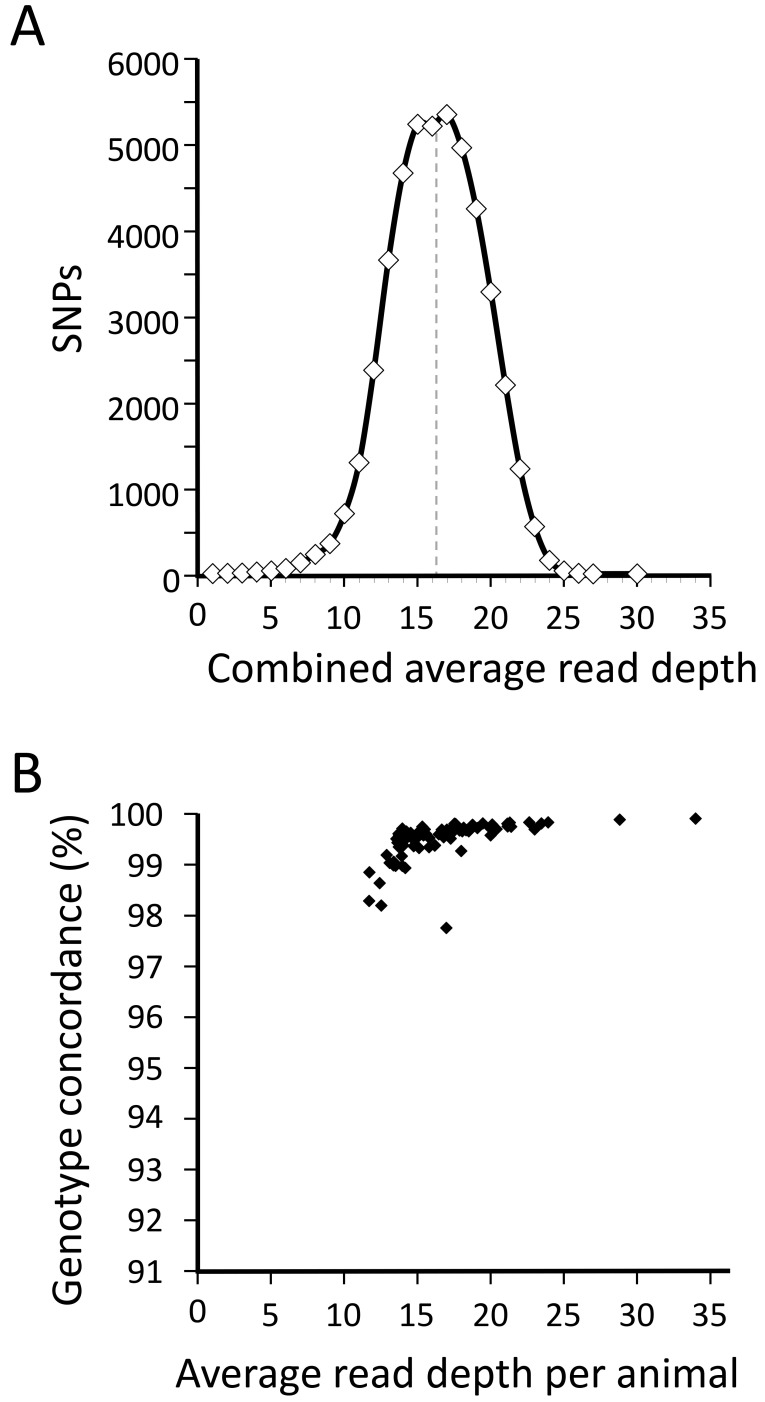
Comparison of WGS genotypes from 96 rams with those from bead arrays. ( **A**) The distribution of average WGS read depth across 45,946 SNP sites for 96 sires combined. ( **B**) A comparison of the average WGS read depth per animal to the average genotype concordance between 45,946 WGS and bead array genotypes.

The coverage and integrity of the WGS datasets were also evaluated at 50,357 evenly distributed, autosomal SNP sites from bead array data ^25^. When plotted as a distribution of read depths by SNPs for all animals combined, the read depth was normally distributed with a mode near 16 ( Figure 3A). The calculated average read depth per SNP per animal was 16.8 for the 50 k bead array SNPs (Min 11.7, Max 34.2, SD 3.5), compared to 17.0 for the 163 reference SNPs above. Averaged over all animals, the concordance between WGS genotypes and those from the bead array was 99.5% ( Figure 3B) compared to 99.4% for the 163 reference SNPs. The genotype concordance reached a maximum at approximately 99.89% for the animal with the highest read depth (34.2-fold, 97.7 GB Q20 data). Taken together, the WGS genotype results for 163 reference SNPs was consistent with those for the 50 k bead array SNPs and indicated that the WGS datasets from these 96 rams are of sufficient quality and coverage for use in identifying and decoding gene variants in U.S. sheep.

### Protein variants encoded by GDF9, BMP15, and BMPR1B genes

**Figure 4.**
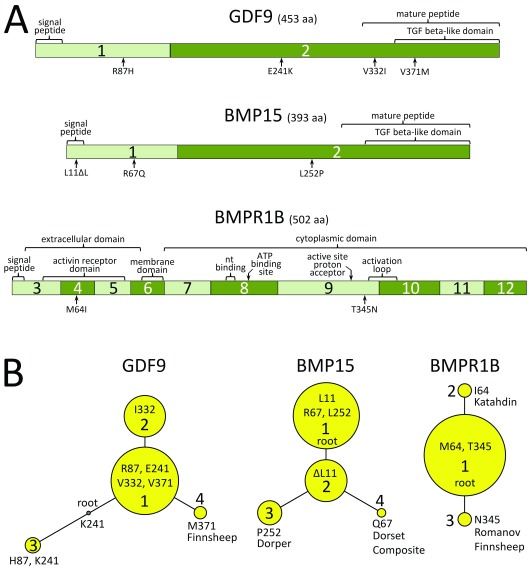
Physical maps and rooted maximum parsimony phylogenetic trees of GDF9, BMP15, and BMPR1B protein variants in U.S. sheep. ( **A**) Physical maps of GDF9, BMP15, and BMPR1B exon and protein domains in relationship to missense variants. ( **B**) Maximum parsimony phylogenetic trees of haplotype-phased protein variant identified in the sheep diversity panel. For each gene analyzed, the most frequent protein isoform was defined as “variant 1” and used as the reference sequence for each tree. Each node in a tree represents a different protein isoform that varies by one amino acid compared to adjacent nodes. The areas of the circles are proportional to the variant frequency in the panel of 96 rams. The trees were rooted based on evolutionary conservation of residues in closely related species. The predicted root of GDF9 was not observed in the 96 rams.

**Figure 5.**
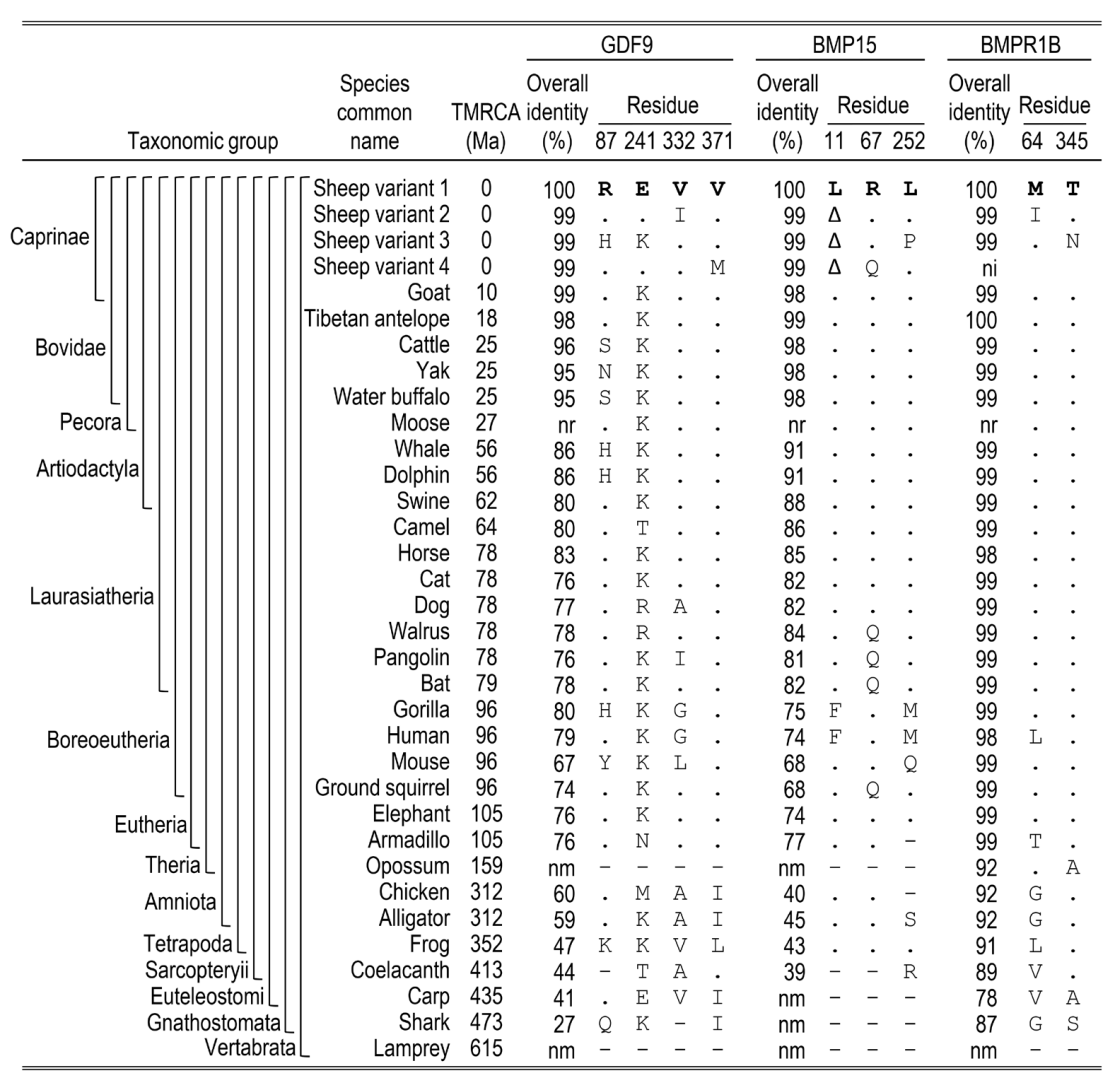
Evolutionary comparison GDF9, BMP15, and BMPR1B protein residues at their variant sites in U.S. sheep. Aligned protein sequences from a representative subset of 29 vertebrate species were compared. Abbreviations and symbols are as follows: TMRCA, estimated time to most recent common ancestor in millions of years ^48^; letters, IUPAC/IUBMB codes for amino acids; dot, amino acid residues identical to those in sheep “variant 1”; triangle, net deletion of one leucine residue in BMP15 positions 10 and 11 where two leucine residues are commonly present; ni, a fourth protein variant was not identified for BMPR1B; nr, not in refseq_protein database and thus residues were determined by analyzing WGS data; dash, not enough sequence similarity for comparison or missing polypeptide region; nm, did not match a refseq_protein in the database for that species.

The WGS data for the 96 rams were used to analyze the coding regions of *GDF9, BMP15,* and *BMPR1B*. These genes encode proteins of 453, 393, and 502 amino acids, respectively, each with multiple functional domains ( Figure 4A). Viewing the aligned sequences and detecting variants was simple, fast, and accurate with the IGV software and a publicly available web-based browser developed for this purpose ( Figure S1, Table S1). Nine missense variants were observed in the three genes with the 96 genomes ( Table 1). Four of the nine variants were not previously reported: *BMP15* (R67Q, L252P) and *BMPR1B* (M64I, T345N). No other missense, nonsense, frameshift, splice site, or indel variants affecting the coding region were detected. A comparative list of the coding variants discovered here is given in Table 2, together with those previously reported for the three genes. Eleven protein sequence isoforms were predicted from phased combinations of codon variants ( Table 3). Haplotypes were translated and placed in the context of a phylogenetic tree for predicted variants for *GDF9, BMP15,* and *BMPR1B* ( Figure 4B). The trees were rooted based on the pattern of evolutionary conservation of the residues in vertebrates ( Figure 5). All four of the previously unreported protein variants encoded by *BMP15* and *BMPR1B* were on the distal nodes of their respective tree, indicating they arose after those on adjacent nodes. The previously reported *GDF9* V371M variant was present in our reference panel only in Finnsheep ( Table 4). Alleles encoding the M371 residue are associated with increased litter size in both carriers and homozygous individuals ( Table 2). The novel *BMP15* R67Q and L252P variants were confined to the Dorset and Dorper breed groups of our reference panel, respectively. The novel *BMPR1B* M64I variant was only present in the Katahdin breed group, while the novel *BMPR1B* N345 variant was observed in both Romanov and Finnsheep breed groups.

**Table 1.** DNA sequence information for *GDF9, BMP15, and BMP15R1B* missense variants identified in the sheep diversity panel (MSDPv2.4).

Gene	Codon variant ^a^	Position (Oar_v3.1)	Exon	Pro. dom. ^b^	Codon ^c^	Codon allele ^d^	MAF ^e^	Flanking genomic sequence
*GDF9*	R87H	chr5:41,843,258	1	PRO	c **R**c	c **G**c = R c **A**c = H	0.037	ggtgggagacacagacctggtctcctttcccctctcttagaggttctgtatgatgggcacggggaaccccccaggctgcagccagatgacagagctttgc **[R]** ctacatgaagaggctctataaggcatacgctaccaaggaggggacccctaaatccaacagacgccacctctacaacactgttcggctcttcaccccctgt
	E241K	chr5:41,841,675	2	PRO	**R**aa	**G**aa = E **A**aa = K	0.037	gaaatacaaatggatggagattgatgtgacggctcctcttgagcctctggtggcctcccacaagaggaatattcacatgtctgtaaattttacatgtgcg **[R]** aagaccagctgcagcatccttcagcgcgggacagcctgtttaacatgactcttctcgtagcgccctcactgcttttgtatctgaacgacacaagtgctca
	V332I	chr5:41,841,402	2	MAT	**R**tt	**G**tt = V **A**tt = I	0.245	atctgcctaccccgtgggagaagaagctgctgagggtgtaagatcgtcccgtcaccgcagagaccaggagagtgccagctctgaattgaagaagcctctg **[R]** ttccagcttcagtcaatctgagtgaatacttcaaacagtttctttttccccagaatgaatgtgagctccatgactttagacttagctttagtcagctgaa
	V371M	chr5:41,841,285	2	MAT	**R**tg	**G**tg = V **A**tg = M	0.026	tctgagtgaatacttcaaacagtttctttttccccagaatgaatgtgagctccatgactttagacttagctttagtcagctgaagtgggacaactggatt **[R]** tggccccacacaaatacaaccctcgatactgtaaaggggactgtcccagggcggtcggacatcggtatggctctccggttcacaccatggtgcagaacat
*BMP15*	L11ΔL ^f^	chrX:50,977,397	1	PRO	indel	cttctt = LL ctt = L	0.344	gtaaaaggaaaggtttaaagcgttatcctttgggcttttatcagaacatgttgctgaacaccaagcttttcaagatggtcctcctgagcatccttagaatc **[cttctt/ctt]** tggggactggtgctttttatggaacatagggtccaaatgacacaggtagggcagccctctattgcccacctgcctgaggcccctaccttgcccctgattc
	R67Q	chrX:50,977,228	1	PRO	c **R**g	c **G**g = R c **A**g = Q	0.010	cacctgcctgaggcccctaccttgcccctgattcaggagctgctagaagaagcccctggcaagcagcagaggaagccgcgggtcttagggcatcccttac **[R]** gtatatgctggagctgtaccagcgttcagctgacgcaagtggacaccctagggaaaaccgcaccattggggccaccatggtgaggctggtgaggccgctg
	L252P	chrX:50,971,365	1	MAT	c **Y**g	c **T**g = L c **C**g = P	0.083	ttctggtggcatggcacttcatcattggacactgtcttcttgttactgtatttcaatgacactcagagtgttcagaagaccaaacctctccctaaaggcc **[Y]** gaaagagtttacagaaaaagacccttctcttctcttgaggagggctcgtcaagcaggcagtattgcatcggaagttcctggcccctccagggagcatgat
*BMPR1B*	M64I	chr6:29,401,381	4	AR	at **R**	at **G** = M at **A** = I	0.026	acacacacacacacacacacacacacacatactttgcctgtttgatctttagcacagatggatattgtttcacgatgatagaagaagatgactctgggat **[R]** cctgtggtcacttctggatgtctaggactagaaggctcagattttcagtgtcgggtaaggaagataccttggttccactttgtaaccttttattggcaag
	T345N	chr6:29,380,795	9	AS-AL	a **M**t	a **A**t = T a **C**t = N	0.026	cctgtggtcacttctggatgtctaggactagaaggctcagattttcagtgtcgggtaaggaagataccttggttccactttgtaaccttttattggcaag **[M]** ctctgtcacttacacactgaaatctttagcactcaaggcaaaccagcaattgcccatcgagatctgaaaagtaagaacatcctggtgaagaaaaatggaa

^a^All variants and sequences are oriented from the sense strand perspective. However, *GDF9, BMP15,* and *BMPR1B* are oriented in the opposite direction with regards to the Oar_v3.1 reference assembly. Alphabetical abbreviations for relevant amino acids: E, glutamate; H, histidine; I, isoleucine; K, lysine; L, leucine; M, methionine; N, asparagine; P, proline; Q, glutamine; R, arginine; T, threonine; and V, valine. ^b^Protein domain abbreviations: PRO, propeptide; MAT, mature peptide; AR, activin receptor domain; and AS-AL, between the active site proton acceptor and the activation loop domains. ^c^IUPAC/IUBMB ambiguity codes used for nucleotides: R = a/g, Y = c/t, M = a/c, K = g/t, S = c/g, W = a/t ^35^. ^d^The major allele is listed first. ^e^Minor allele frequency in MSDPv2.4. ^f^The L11ΔL variant is an abbreviation for p.(Leu10_11delinsLeu), the recommended nomenclature for this variant by the Human Genome Variation Society ^36^.

**Table 2.** Comparison of missense, nonsense, and frameshift variants in the coding sequences of ovine *GDF9, BMP15, BMPR1B* and their phenotypic association in sheep.

Gene (Chr.)	Coding variant ^a^	Phenotype	Breed groups	Ref.
GDF9 (Chr5)	R87H	None reported	Multiple	37, this work
	E241K	None reported	Multiple	37, 38, this work
	R315C	Fecundity, Vacaria, *FecG ^V^*	Ile de France	39
	V332I	None reported	Multiple	37, 38, this work
	F345C	Fecundity, Embrapa, *FecG ^E^*	Santa Inês	40
	V371M	Fecundity	Finnish landrace	11, 26, 37, this work
	S395F	Fecundity, High Fertility, *FecG ^H^*	Belclare, Cambridge	37
	S427R	Fecundity, Thoka, *FecT ^T^*	Icelandic	41
BMP15 (ChrX)	L11ΔL	None reported	Multiple	37, this work
	**R67Q**	**Unknown, Dorset**	**Dorset**	**This work**
	W154Δ17	Fecundity, Rasa Aragonesa, *FecX ^R^*	Rasa Aragonesa	42, 43
	Q239stop	Fecundity, Galway, *FecX ^G^*	Belclare, Cambridge	37
	**L252P**	**Unknown, Dorper**	**Dorper**	**This work**
	Q291stop	Fecundity, Hanna, *FecX ^H^*	Romney	44
	V299D	Fecundity, Inverdale, *FecX ^I^*	Romney	44
	T317I	Fecundity, Grivette, *FecX ^GR^*	Grivette	45
	C321Y	Fecundity, Lacaune, *FecX ^L^*	Lacaune	46
	N337H	Fecundity, Olkuska, *FecX ^O^*	Olkuska	45
	S367I	Fecundity, Belclare, *FecX ^B^*	Belclare	37
BMPR1B (Chr6)	**M64I**	**Unknown, Katahdin**	**Katahdin**	**This work**
	Q249R	Fecundity Booroola, *FecB ^B^*	Booroola, *et al.*	14, 29, 33, 47
	**T345N**	**Unknown, Romanov**	**Romanov**	**This work**

^a^Bold font indicates previously unreported variants affecting the protein sequence.

**Table 3.** Frequencies of haplotype-phased GDF9, BMP15, and BMPR1B protein variants in U.S. sheep.

Protein	Protein variant code	Variant amino acids ^a^	Sheep diversity panel (n = 96) ^b^
GDF9	1	R87, E241, V332, V371	0.693
	2	R87, E241, **I332**, V371	0.245
	3	**H87**, **K241**, V332, V371	0.036
	4	R87, E241, V332, **M371**	0.026
BMP15	1	L11, R67, L252	0.656
	2	**ΔL**, R67, L252	0.250
	3	**ΔL**, R67, **P252**	0.083
	4	**ΔL**, **Q67**, L252	0.010
BMPR1B	1	M64, T345	0.948
	2	**I64**, T345	0.026
	3	M64, **N345**	0.026

^a^The bolded residues are those differing from “variant 1” in each gene. ^b^The protein variant frequency.

**Table 4.** Frequency estimates of GDF9, BMP15, and BMPR1B protein variants by breed group.

		Protein variant frequency ^a^												
		GDF9					BMP15					BMPR1B		
		1	2	3	4 ^b^		1	2	3 ^c^	4 ^c^		1	2 ^d^	3 ^d^
**Breed group**	No.	(Ref.)	(I332)	(H87, K241)	(M371)		(Ref.)	(ΔL)	(P252)	(Q67)		(Ref.)	(I64)	(N345)
Dorper	6	0.33	0.67	- ^e^	-		0.17	0.50	0.33	-		1.00	-	-
Dorper, white	4	0.75	0.25	-	-		-	-	1.00	-		1.00	-	-
Dorset	11	0.64	0.36	-	-		0.82	0.09	-	0.09		1.00	-	-
Finn	10	0.55	0.20	-	0.25		0.70	0.30	-	-		0.95	-	0.05
Katahdin	8	0.75	0.25	-	-		1.00	-	-	-		0.81	0.19	-
Navajo-Churro	1	1.00	-	-	-		1.00	-	-	-		1.00	-	-
Rambouillet	10	0.85	0.15	-	-		0.20	0.80	-	-		1.00	-	-
Romanov	10	0.30	0.60	0.10	-		0.30	0.70	-	-		0.80	-	0.20
Suffolk	9	0.94	0.06	-	-		1.00	-	-	-		1.00	-	-
Texel	10	0.70	0.10	0.20	-		0.90	0.10	-	-		1.00	-	-
Composite	17	0.88	0.09	0.03	-		0.82	0.06	0.12	-		0.94	0.06	-

^a^The variants correspond to those shown in Figure 4. The distinctive missense variant or reference isoform is indicated in parentheses. ^b^GDF9 protein “variant 4" contains the M371 amino acid previously associated with litter size in Finnish landrace sheep ^11, 26, 37^. ^c^BMP15 protein “variants 3 and 4" contain the previously unreported P252, and Q67 residues, respectively. ^d^BMPR1B protein “variants 2 and 3” contain the previously unreported I64 and N345 missense variants, respectively. ^e^Hyphen indicates the variant was not detected in that group.

An analysis of amino acid sequence conservation among species helped identify critical residues more likely to be involved in important protein functions. Ovine GDF9, BMP15 and BMPR1B were 80, 88, and 99% identical to other Artiodactyla species, at the propeptide sequence level ( Figure 5). We predict that variant residues in highly conserved protein domains are more likely to affect ovulation rate and litter size. The most well conserved residue in GDF9 showing missense variation among sheep breeds is V371, located in the TGF-β-like domain. Throughout Eutheria, sheep were the only species observed to have the M371 residue encoded by *GDF9* ( Figure 5). This is consistent with the substitution of the M371 residue causing reduced protein function, and therefore increased litter size in Finnish Landrace sheep ( Table 2). Less conserved were the *GDF9* residues at positions 87, 241, and 332, which are variable throughout Eutheria species and have not been associated with fecundity in sheep. With regards to missense variants in the other TGF-β ligand, BMP15 residues at positions 11, 67, and 252, were conserved through most of the Laurasiatheria, although the L11 deletion variant is common in sheep and has not been associated with fecundity ( Table 2). Since Q67 and P252 substitutions in *BMP15* have not been previously reported, their impact on protein function or reproductive phenotype has yet to be determined.

Conservation in the TGF-β receptor ligand receptor, BMPR1B, is particularly striking with 98% propeptide identity observed throughout Eutheria species, compared to approximately 76% and 77% for GDF9 and BMP15, respectively. Moreover, BMPR1B residues at positions 64 and 345 are also conserved throughout the Eutheria, suggesting that the I64 and the N345 substitutions in sheep may affect protein function. The I64 substitution in Katahdin sheep is in the extracellular activin receptor domain, whereas the N345 substitution in Romanov sheep is between the active site proton acceptor domain and the activation loop of the cytoplasmic domain ( Figure 4A). Although intriguing, the potential effects of the observed substitutions encoded by *GDF9*, *BMP15* and *BMPR1B* in U.S. Sheep are unknown.

### Retrospective analysis of litter size in daughters of carrier rams

The potential effects of the observed *GDF9*, *BMP15* and *BMPR1B* variants on reproductive phenotypes were examined by analyzing lambing records from daughters of the rams sequenced in this project. There were no database records for daughters of the five Finnsheep rams carrying the *GDF9* allele encoding the M371 residue (i.e., “Variant 4”, Table S2). There were, however, records for 403 daughters sired by eight rams with at least one of the four *BMP15* or *BMPR1B* variants. Together, the eight rams sired 480 lambs in various flocks in seven years, although not all variant genotypes were frequent in these rams ( Table S3– Table S6). Analyses of these data did not reveal a significant correlation between litter size and any of the four *BMP15* or *BMPR1B* variants (95% confidence interval). However, this simple test for association lacked power, and could only detect litter size effects. It remains possible that a well-designed, prospective genetic study may detect biologically and economically relevant differences associated with these variants of highly-conserved residues in developmentally important genes.

## Discussion

We created a searchable and publicly viewable online genomics resource consisting of 96 individuals representing a broad cross section of U.S. sheep breeds, and demonstrated its use for identifying protein variants. The DNA for these 96 rams, together with their 95 tetrad families, is also available for confirming segregation alleles identified in the WGS ^16^. A minimum of 40 GB of short read, paired-end DNA sequence data provided at least 11-fold mapped genome coverage for each animal. The aligned sequences were made available for downloading or viewing online with a customized IGV visualization software that supports accurate manual assessment of gene-specific genetic variation. The average coverage of the sheep diversity panel was 16.8-fold and resulted in an average genotype accuracy of approximately 99.5%. These numbers were consistent with previous results obtained with 96 beef bulls ^9^. This online resource provides the ability to readily inspect gene variants reported in one breed, evaluate them in other breeds, and search for any additional variants that may affect protein structure. The ability to identify the full range of protein variants in a population is critical for designing studies intended to test a candidate gene’s influence on a trait.

The web-based platform worked well for analyzing three ovine genes with previously documented missense variants affecting ovulation rate and litter size. In a matter of hours, each gene was evaluated for any obvious coding variants, scored in the group of 96 rams, and compared to the previously known variants. Of the 14 known causative variants affecting litter size in sheep, only one was observed in the 96 U.S. rams, and only in the Finnsheep breed ( *GDF9* V371M). This is consistent with reports that the highly prolific Finnish Landrace sheep are thought to be the source of the V371M variant ^11, 26^. With regards to U.S. Finnsheep, the frequency of the *GDF9* V371M variant was 0.25%, with five of the 10 rams having zero copies of the V371M variant. Since ewes homozygous for the M317 variant are known to be fertile, there is a good opportunity for breeders to modulate the frequency of the *GDF9* V371M variant in their purebred Finnsheep flocks, and thereby attain a more optimal litter size for their ewes.

The WGS analysis also revealed four previously unreported missense variants: *BMP15* R67Q and L252P; *BMPR1B* M64I and T345N. Although our preliminary tests for association between variants and litter size did not detect a significant difference, the evidence for dismissing these candidates is not compelling due to the limited number of sires with the variant allele. In spite of having no direct evidence of phenotypic effects associated with these alleles, analysis of the evolutionary conservation of residues at variant sites, their locations within the protein domains, and the effects on ovulation in other species has provided some insight. For example, the *BMP15* R67Q variant found in Dorset was the least conserved, and predicted to be the least likely to affect function among placental mammals. Since the Q67 residue is present in several other Eutheria, and is not part of the mature BMP15 peptide ligand, its occurrence would seem to be a functional evolutionary option ( Figure 5). In humans, the equivalent variant (R68Q) was reported in the 1000 Genomes Project with no apparent disease effect noted (rs782187019) ^27^. However, a tryptophan (W) substitution at this same position in humans causes premature ovarian failure and primary ovarian insufficiency (i.e., R68W) ^28^. Thus, some substitutions at this position may cause loss of function in some mammals, but it appears as though Q67 may not be one of them.

Unlike the R67Q variant, the L252P variant encoded by *BMP15* was not observed in any other vertebrate species and was strictly conserved throughout the Laurasiatheria species. The P252 residue does not appear in the mature BMP15 peptide, however, it is plausible that the non-conservative substitution of P252 for L252 could interfere with post-translational processing of the mature peptide. In primate species, M253 is the equivalent residue to ovine position L252P, and healthy human individuals represented in the 1000 Genomes Project have rare heterozygous substitutions of V253 and T253 with no pathology reported. Because alleles with the P252 residue were present at a high frequency (1.0 in four White Doper), it’s unlikely that the homozygous state causes sterility in ewes. However, the possibility remains that P252 residue may decrease function, and that two copies of a slightly less functional BMP15 may increase the ovulation rate and litter size.

In contrast to the numerous missense variants encoded by the ovine *GDF9* and *BMP15* genes, there has been only one missense variant identified in the receptor gene, *BMPR1B* (Q249R). This variant was first discovered in Booroola Merino sheep ^14, 29^, and subsequently reported in Garole ^30^, Javanese ^30^, Chhotanagpuri ^31^, Iranian Kalehkoohi ^32^, small-tailed Han ^33^, Hu and Chinese Merino ^34^ sheep. In the present report, we did not observe the Q249R variant in any of the WGS from 96 U.S. sheep. Rather, two previously unrecognized *BMPR1B* variants were identified: M64I and T345N. The M64I variant was present in two of eight Katahdin rams (including a homozygote), and two of 17 composite rams containing Suffolk, Colombia and Hampshire germplasm. The I64 substitution was not present in other vertebrate protein sequences and was conserved throughout the Theria with the notable exception of humans, manatees, and armadillos. No variants have been reported in the 1000 Genomes Project for the equivalent position in humans. The M64I variant is positioned in the extracellular activin receptor domain, whose function is to bind ligands for receptor activation. It is plausible that the enhanced fertility and prolificacy, which the Katahdin breed is known for, is conferred in part by this variant.

The second *BMPR1B* variant, T345N, is located inside the cell between two closely spaced active site domains and was present in three of ten Romanov rams (including a homozygote), and one of ten Finnsheep rams. The T345 residue is conserved throughout Tetrapoda species and N345 was not found in any Vertebrata species. A search for human variants in the 1000 Genomes Project revealed only a rare S345 substitution with no pathology reported. Based on the location of the T345 variant near the active site, its strict evolutionary conservation in vertebrates, and that it was found in the two most prolific U.S. breeds, we hypothesize that the N345 residue diminishes the function of the BMPR1B receptor and may influence ovulation and litter size. The *BMPR1B* T345N variant thus represents a high-priority candidate allele for validation studies in these breeds. If any of these newly discovered variants are confirmed to be associated with litter size, DNA-based tests for them could be incorporated into existing genetic testing platforms and used to select for important traits and manage production. Since the number of lambs produced per ewe per year is of fundamental economic importance to sheep production regardless of the production system, these types of DNA tests would be helpful for producers interested in modulating lamb production to match available resources and maintain long-term sustainability.

## Conclusion

In summary, the WGS resources described here are suitable for use in identifying and decoding gene variants in the vast majority of U.S. sheep. When applied to *GDF9, BMP15* and *BMPR1B* genes, the findings suggest there may be variants circulating in the U.S. that could be further evaluated for potential use to increase litter size in U.S. breeds. These resources, including the web interface, underlying sequence data, and the associated information are available to researchers, companies, veterinarians, and producers for use without restriction.

## Data availability

The data referenced by this article are under copyright with the following copyright statement: Copyright: © 2017 Heaton MP et al.

Validated sheep FASTQ files are available in the NCBI SRA under accession numbers SRX2185832-SRX2185868; SRX2185872-SRX2185977; SRX2186010-SRX2186189; SRX2186191-SRX2186294; SRX2186381-SRX2186766; SRX2186768-SRX2186784; SRX2186786-SRX2186798; SRX2186800-SRX2186879.

The data have also been deposited with links to BioProject accession number PRJNA324837 in the NCBI Bio-Project database.

In addition, access to the aligned sequences is available via USDA internet site: http://www.ars.usda.gov/Services/Docs.htm?docid=25585. Download access to the BAM files is available at the Intrepid Bioinformatics site:

http://server1.intrepidbio.com/FeatureBrowser/customlist/record?listid=7918711123

Lambing records for daughters of carrier rams were retrieved from the USMARC historical database, which is not accessible to the public. Table S3– TableS6 provide summary data from these records, which is adequate for the reproducibility and re-analysis purposes of this article.

## Supplementary material

**Table S1.** GDF9, BMP15, and BMPR1B genotypes recorded manually from WGS reads mapped to OAR_v3.1 assembly for the USMARC Sheep Diversity Panel v2.4.

**Table S2.** Haplotype-phased genotypes (diplotypes) for *GDF9*, *BMP15*, and *BMPR1B* genes in the MSDPv2.4.

**Table S3.** Effect of sire copies of *BMP15* P252 alleles (“Variant 3”) on daughter litter size.

**Table S4.** Effect of sire copies of *BMP15* Q67 alleles (“Variant 4”) on daughter litter size.

**Table S5.** Effect of sire copies of *BMPR1B* I64 alleles (“Variant 2”) on daughter litter size.

**Table S6.** Effect of sire copies of *BMPR1B* N345 alleles (“Variant 3”) on daughter litter size.

**Figure S1.** Screen image of Integrated Genome Viewer (IGV) software displaying *GDF9* V332I genotype data for eight sheep.

**Supplementary File 1.** VCF file of 50,357 SNP variants used in comparing WGS genotypes to those from the OvineSNP50 bead array.
