# Supplementary figures and images for: Using sheep genomes from diverse U.S. breeds to identify missense variants in genes affecting fecundity
Source: F1000Res. 2017 Aug 2;6:1303. [Version 1] doi: 10.12688/f1000research.12216.1 (PMC5590088; doi:10.12688/f1000research.12216.1)

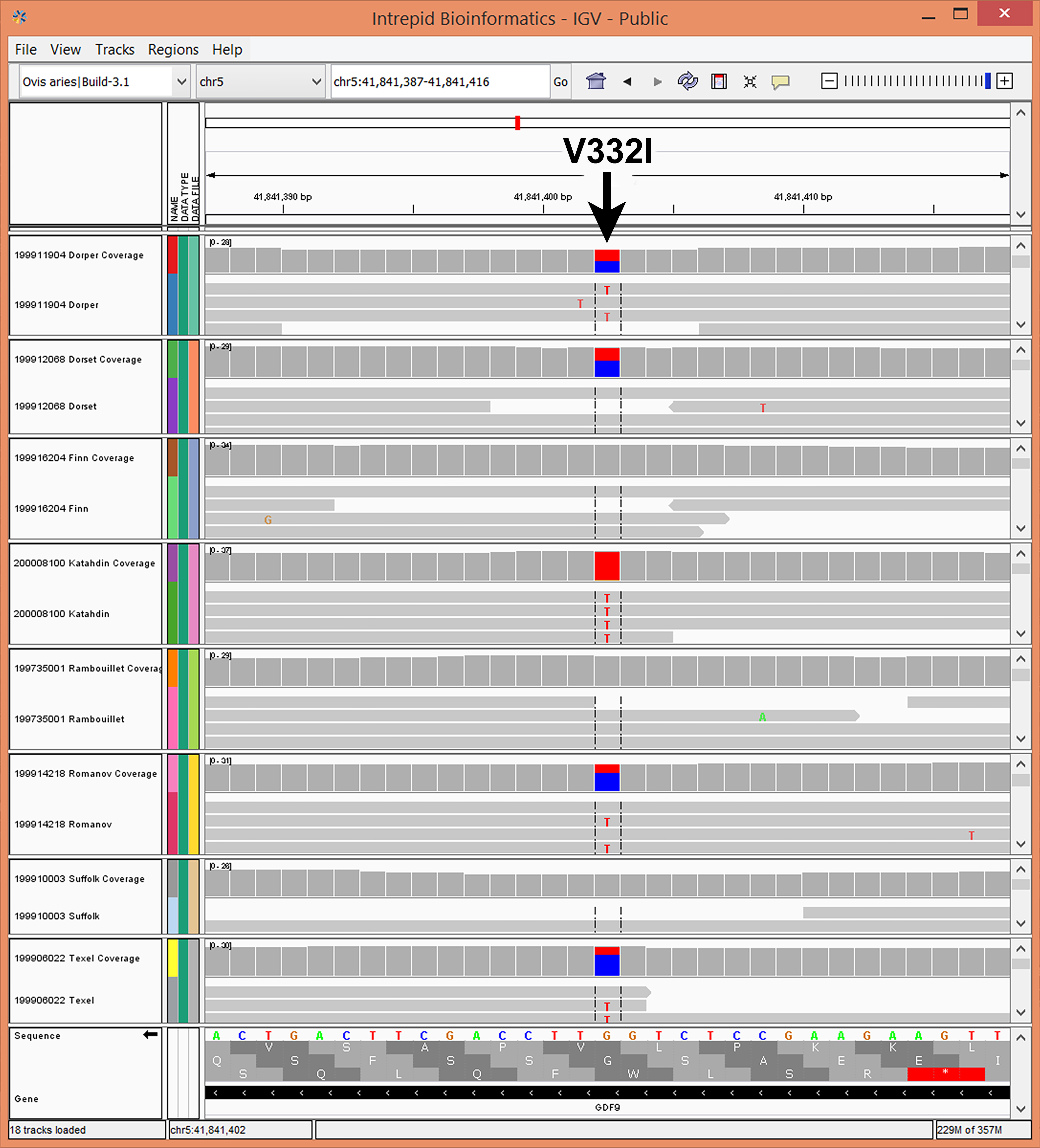

Supplement: Supplementary file 7 [file f1000research-6-13224-s0006.tgz › c3451aab-ee2a-43fe-8a58-df9cf7220c11.tif]
