# Supplementary material for: Using sheep genomes from diverse U.S. breeds to identify missense variants in genes affecting fecundity
Source: F1000Res. 2017 Aug 2;6:1303. [Version 1] doi: 10.12688/f1000research.12216.1 (PMC5590088; doi:10.12688/f1000research.12216.1)
Supplement: Peer review history

# Peer review: Referee response for version 1

**DOI:** 10.5256/f1000research.13224.r25465

**Published:** 2017-09-06

**Authors:** Eyal Seroussi

**Affiliations:** 1 Institute of Animal Science, Agricultural Research Organization (ARO), Rishon LeZion, Israel

       The main aim of the publication entitled “Using sheep genomes from diverse U.S. breeds to identify missense variants in genes affecting fecundity” is to establish a publicly accessible resource of sequenced genomes that represent popular U.S. sheep breeds. A key factor for the usefulness of such resource is the fold coverage. While in Introduction a “16x WGS resource” is mentioned, in Results it is indicated that: “the average calculated read depth (17.0)”, the submissions to the SRA database are all entitled “12x WGS of USMARC Sheep…” and the author’s website referred in the manuscript: “USDA, ARS, USMARC internet site” is entitled: “10x WGS of …”. The latter number would have indicated a limited utility for Genotyping-By-Sequencing (GBS) of SNPs, as the typical coverage is not evenly distributed along the genome, and many loci would lack the minimal coverage of 5x. This coverage allows detecting homozygotes with less than a 0.05% uncertainty, assuming ideal conditions of a 0.5 probability to detect an allele and no sequencing errors. Practically, detection rate is biased and sequence errors do exist. Therefore, the authors put much effort to estimate the rate of genotyping errors by comparing their genotypes with bead array data, concluding that no animals had a SNP genotype accuracy of less than 97%. Since this calculation of error rate involves errors introduced by both genotyping methods, a more straightforward approach can be considered by analyzing non-autosomal loci on chromosome X; there all genotypes must be homozygous. Table S1 offers such a possibility for the *BMP15* gene; where two heterozygotes were encountered out of 288 genotypes, which indicates that either sequencing errors or contaminations may introduce 0.7% of the error.

                 Indeed, sequence cross contamination is a known problem of sequenced genomes ^1^; and sequencing projects should be routinely controlled for DNA contaminants. As the authors did not refer to this problem, I tested one of their 923 submissions that has a median size (4.6 G bases, SRX2186704) by analyzing the sequence reads that do not map to the sheep genome (Oar_v4). Using the *GAP5* software ^2^, the reads sent to the failures.seq file were de-novo assembled into contigs using *MIRA4* sequence assembler ^3^. As the current genome version lacks the Y chromosome, most of these contigs were similar to submissions of Y chromosome orthologs of other ruminants; yet, several contigs resembled the *Onchocerca flexuosa *genome, the largest of which was of 3372 bp and had 502 reads with 83% identity to this worm genome. This suggests that this individual was infected by a parasitic roundworm similar to the species that infect red deer, and that the authors present a valuable resource that is important for parasitology ^4^. As I encountered no other DNA contaminants, it is likely that the data presented by the authors is solid and of the highest quality and that the worm DNA was extracted from this animal’s blood.

                 As for identifying missense variants in 3 genes affecting fecundity, I conclude that the authors left no stone unturned to ensure the validity of their genotypes. E. g., Table S1 indicates that the unique homozygous genotype for *BMPR1B* (individual 200117552) had coverage of 41x fold, suggesting that sequence coverage was increased for this individual to ensure this result. Yet, the use of modern tools for predicting the functional effect of amino acid substitutions ^5^ should have warned them that this variation (T345N) is not likely to produce a phenotype (PROVEAN score = -2.191, Neutral). In this respect, the 4 novel variations described in the fecundity genes are of minor importance. Nevertheless, the observation that none of the U.S. popular breeds carries the Booroola mutation should have an impact, as introgression of this mutation revolutionized sheep production in Spain and Israel ^6^. Despite the apparent weakness of the work in identifying important novel variants of fecundity genes, I approve this work as a valuable genomic resource. The authors are advised to control for DNA contaminants, to avoid the discrepancies described by extending the clear and accurate presentation of this work to the affiliated webpages and to discuss the issues raised by this review.

I have read this submission. I believe that I have an appropriate level of expertise to confirm that it is of an acceptable scientific standard.

## References

[1] GruberK: Here, there, and everywhere: From PCRs to next-generation sequencing technologies and sequence databases, DNA contaminants creep in from the most unlikely places. *EMBO Rep*.2015;16(8) : 10.15252/embr.201540822 898-901 10.15252/embr.201540822 26150097PMC4552482

[2] BonfieldJKWhitwhamA: Gap5--editing the billion fragment sequence assembly. *Bioinformatics*.2010;26(14) : 10.1093/bioinformatics/btq268 1699-703 10.1093/bioinformatics/btq268 20513662PMC2894512

[3] ChevreuxBPfistererTDrescherBDrieselAJMüllerWEWetterTSuhaiS: Using the miraEST assembler for reliable and automated mRNA transcript assembly and SNP detection in sequenced ESTs. *Genome Res*.2004;14(6) : 10.1101/gr.1917404 1147-59 10.1101/gr.1917404 15140833PMC419793

[4] LopesRJMéridaAMCarneiroM: Unleashing the Potential of Public Genomic Resources to Find Parasite Genetic Data. *Trends Parasitol*.2017; 10.1016/j.pt.2017.06.006 10.1016/j.pt.2017.06.006 28716403

[5] ChoiYSimsGEMurphySMillerJRChanAP: Predicting the functional effect of amino acid substitutions and indels. *PLoS One*.2012;7(10) : 10.1371/journal.pone.0046688 e46688 10.1371/journal.pone.0046688 23056405PMC3466303

[6] SeroussiERosovAShirakALamAGootwineE: Unveiling genomic regions that underlie differences between Afec-Assaf sheep and its parental Awassi breed. *Genet Sel Evol*.2017;49(1) : 10.1186/s12711-017-0296-3 19 10.1186/s12711-017-0296-3 28187715PMC5301402

# Peer review: Referee response for version 1

**DOI:** 10.5256/f1000research.13224.r24897

**Published:** 2017-08-29

**Authors:** Christine Couldrey

**Affiliations:** 1 Research and Development, Livestock Improvement Corporation, Hamilton, New Zealand

The manuscript entitled “Using sheep genomes from diverse U.S. breeds to identify missense variants in genes affecting fecundity” provides an informative overview of a publicly searchable DNA sequence resource for U.S. sheep that the authors have generated. The manuscript is well written.

 The manuscript is largely descriptive in nature but does include a proof of principal type study to highlight the potential usefulness of this dataset.  The study is appropriately designed and technically sound. Most of the conclusions drawn are adequately supported by the results. Where the authors have not identified phenotypic effects of amino acid changes they have acknowledged that this study does lack power to make definitive statements.

 A few details could do with further detail/clarification/some modification:

1. In the last paragraph on page 4 where the manuscript states “when the accuracy of the WGS genotypes exceeded 97%”…… Please explain why 97% was chosen as the threshold.      
2. Page 5 under heading Identifying protein variants encoded by GDF9, BMP15, and BMPR1B: the methods describe haplotype phasing, however, it is unclear as to how many of the 96 sheep were able to be phased and used in analysis. It is therefore difficult to determine the validity of this method. The methods or corresponding results section should be expanded to include this information. 
3. Page 5 Methods section under heading Statistical analysis of litter size in daughters of carrier rams:  Please include information of how many rams had daughter lambing records for each breed and each of the variants identified 
4. Page 6 The authors should include some discussion/information about the inclusion of animals that had the lowest concordances with other genotyping platforms, particularly the animals with ~17X coverage and a concordance of ~97%.  In some research facilities this (and some of the other animals with greater than 10X coverage and less than 99% concordance) would be treated as suspect and excluded, or further analysis undertaken - if the latter is the case, please include the further analysis. 
5. Page 7 the sentence “Alleles encoding the M371 residue…” refers to Table 2, however this residue is not referred to in Table 2.  Please correct. 
6. Page 7 “We predict that variant residues in highly conserved protein domains are more likely to affect ovulation rate and littler size”.  While this sentence could be true, it is also possible that protein domains are highly conserved for functions other than ovulation rate and litter size. 
7. Page 12 There is insufficient data to really make the statement “Thus some substitutions at this position may cause loss of function in some mammals but it appears as though Q67 may not be one of them”.  Given that the phenotypes around ovarian failure in sheep are likely not well recorded given the culling of sheep relatively early in life.

I have read this submission. I believe that I have an appropriate level of expertise to confirm that it is of an acceptable scientific standard.
